# Development of Universal Primer Sets for Zika Virus Envelope Gene Amplification and Sequencing

**DOI:** 10.3390/ijms27188017

**Published:** 2026-09-09

**Authors:** Léo Shigueki Sato, Yuki Tayama, Gabriel Gazzoni Araújo Gonçalves, Luiz Carlos Alves, Fábio André Brayner, Shangfan Hu, Mya Myat Ngwe Tun, Arata Hidano, Yuki Takamatsu

**Affiliations:** 1 Department of Virology, Institute of Tropical Medicine (NEKKEN), Nagasaki University, Nagasaki 852-8523, Japan; sshiguekisato@gmail.com (L.S.S.); yuki-tyma@nagasaki-u.ac.jp (Y.T.); hushangfan@nagasaki-u.ac.jp (S.H.); arata.hidano@nagasaki-u.ac.jp (A.H.); 2 Graduate School of Biomedical Sciences, Nagasaki University, Nagasaki 852-8523, Japan; 3 Brazil Research Station, National Research Center for the Control and Prevention of Infectious Diseases (CCPID), Nagasaki University, Nagasaki 852-8523, Japan; 4 Keizo Asami Institute (iLIKA), Federal University of Pernambuco (UFPE), Recife 50670-901, PE, Brazil; gabrielgazzoni@hotmail.com (G.G.A.G.); luizcarlos.alves@fiocruz.br (L.C.A.); fabio.brayner@fiocruz.br (F.A.B.); 5 Department of Parasitology, Aggeu Magalhães Institute (FIOCRUZ-PE), Recife 50740-465, PE, Brazil; 6 Department of Tropical Viral Vaccine Development, Institute of Tropical Medicine (NEKKEN), Nagasaki University, Nagasaki 852-8523, Japan; ntmyamyat@nagasaki-u.ac.jp; 7 Center for Vaccines and Therapeutic Antibodies for Emerging Infectious Diseases, Shimane University, Izumo 693-0021, Japan

**Keywords:** Zika virus, envelope gene, RT-PCR, sequencing, lineage

## Abstract

The Zika virus (ZIKV) remains an important public health concern owing to its association with neurological disorders and congenital abnormalities. Genetically, ZIKV is classified into two major lineages—African and Asian—and continues to evolve, with evidence of genetic diversification following its introduction into the Americas. The study of the envelope (E) gene of ZIKV is a key tool for understanding viral biology, phylogenetic analysis, and developing antiviral drugs and vaccine candidates. However, few studies have focused on designing primers capable of efficiently detecting and amplifying this essential gene. In this study, we developed universal primer sets capable of amplifying and sequencing the full-length ZIKV E gene across genetically diverse lineages and strains. Primers were designed based on representative ZIKV sequences available in public databases and validated using viral isolates and in vitro-spiked plasma samples. The assay demonstrated high specificity and analytical sensitivity, with the analytical detection limit established using serially diluted DNA amplicons from the target region. Furthermore, ZIKV was successfully detected in plasma samples spiked at 1 × 10^3^ PFU/mL, which enabled complete sequencing of the E gene. These findings highlight the utility of our primers for molecular epidemiological studies, strengthening arbovirus surveillance and improving diagnostic capacity in regions where multiple arboviruses co-circulate, using a simple and reliable assay.

## 1. Introduction

The Zika virus (*Orthoflavivirus zikaense*, ZIKV) is an arbovirus that has emerged as a significant public health concern worldwide because of its association with neurological disorders and congenital abnormalities. ZIKV is an enveloped, positive-sense single-stranded RNA virus belonging to the *Orthoflavivirus* genus of the *Flaviviridae* family [1,2,3]. Its genome is approximately 10.8 kb in length and comprises 10 protein-encoding genes, including three structural proteins [envelope (E), membrane (M), and capsid (C)], which are fundamental for viral structure formation, and seven non-structural proteins (NS) (NS1, NS2A, NS2B, NS3, NS4A, NS4B, and NS5), which play a critical role in the replication and assembly of new viruses [1,4,5].

Since its discovery in 1947 in Uganda [6], sporadic human infections have been reported [7]; however, ZIKV received limited epidemiological attention until large outbreaks occurred in the Pacific and the Americas [8]. The virus received global attention in 2016 when the World Health Organization (WHO) declared a Public Health Emergency of International Concern (PHEIC) following an unprecedented increase in microcephaly cases and other manifestations of Congenital Zika Syndrome (CZS) among newborns of ZIKV-infected women in Brazil [9,10,11]. Although most infections are asymptomatic or self-limiting, symptomatic patients typically present with nonspecific manifestations, including fever, rash, conjunctivitis, arthralgia, myalgia, and headache, which overlap considerably with those caused by other arboviruses, such as dengue virus (DENV) and chikungunya virus (CHIKV), making accurate laboratory diagnosis essential for appropriate clinical management and epidemiological surveillance [12,13,14,15,16].

Genetically, ZIKV is classified into two major lineages, African and Asian, which differ by approximately 12% across the coding regions [7,17]. Following the introduction of the Asian lineage into the Americas, continued viral evolution has resulted in genetically diverse circulating strains [8,17,18]. This genetic diversity is epidemiologically relevant because it may compromise the performance of molecular assays designed using a limited number of reference genomes [19,20]. Moreover, because ZIKV frequently co-circulates with other medically important arboviruses that cause clinically similar symptoms, molecular assays must provide high analytical specificity to ensure accurate laboratory diagnosis. Therefore, molecular assays capable of reliably detecting genetically diverse ZIKV strains while maintaining high specificity against closely related flaviviruses are essential for the downstream molecular characterization and accurate laboratory diagnosis of ZIKV infections.

The ZIKV E gene, approximately 1.5 kb in length [21], encodes the major viral surface glycoprotein and is one of the most informative genomic regions for molecular characterization of ZIKV [22]. It contains sufficient sequence variability to discriminate between viral lineages, making it an excellent marker for targeted sequencing [1]. Complete E gene sequences have been widely applied for lineage assignment, phylogenetic reconstruction, identification of emerging variants, and investigation of viral evolution and transmission dynamics [18,23,24,25]. Consequently, reliable amplification and sequencing of the E gene are essential not only for accurate virus detection but also for future molecular epidemiological investigations and genomic surveillance of this virus.

Whole-genome sequencing (WGS) provides the highest resolution for investigating viral evolution and transmission dynamics. However, its routine implementation remains limited in many laboratories because of high costs, specialized infrastructure, and complex bioinformatic pipelines [26]. Consequently, PCR-based amplification followed by Sanger sequencing remains a practical and cost-effective alternative to targeted genome sequencing [27]. Despite the widespread use of whole E gene sequences for molecular epidemiology, to the best of our knowledge, only one previous study has reported primer sets for amplification and sequencing of the entire gene, which were designed using a limited number of reference sequences and evaluated using viruses from a restricted geographic region [25]. Furthermore, previous studies have shown that genetic variability among circulating ZIKV strains may affect PCR performance, underscoring the need for universal primer sets capable of reliably amplifying genetically diverse viruses [24,28,29]. Therefore, this study aimed to develop and validate universal primer sets for the amplification and sequencing of the entire ZIKV E gene using a comprehensive dataset representing the genetic diversity of both the African and Asian ZIKV lineages.

## 2. Results

### 2.1. Primers Information

**Figure 1:**
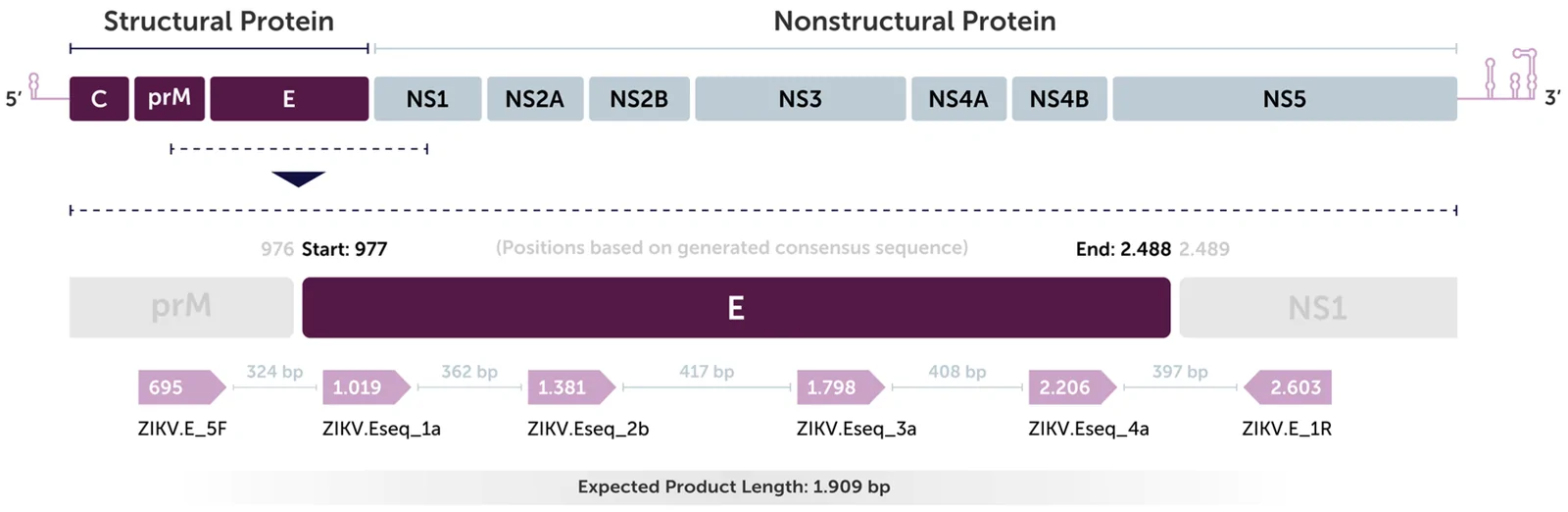
Schematic representation of primer-binding sites in the ZIKV genome. C, Capsid gene; prM, Pre-membrane gene; E, Envelope gene; NS, Non-structural protein gene; bp, base pairs.

For primer design, all 926 ZIKV genome sequences available in GenBank were retrieved. Subsequently, 135 representative sequences were selected using CD-HIT-EST (v4.8.1) [30] at a 97% sequence identity threshold and aligned using MAFFT (v7.151) [31]. The accession numbers of the 135 representative sequences used for primer design are listed in Table S1. Primer sets were designed based on the resulting consensus sequence and evaluated by in silico PCR. The expected product lengths and binding sites of each primer are illustrated in Figure 1.

### 2.2. Viral Detection and Specificity Analysis

**Figure 2:**
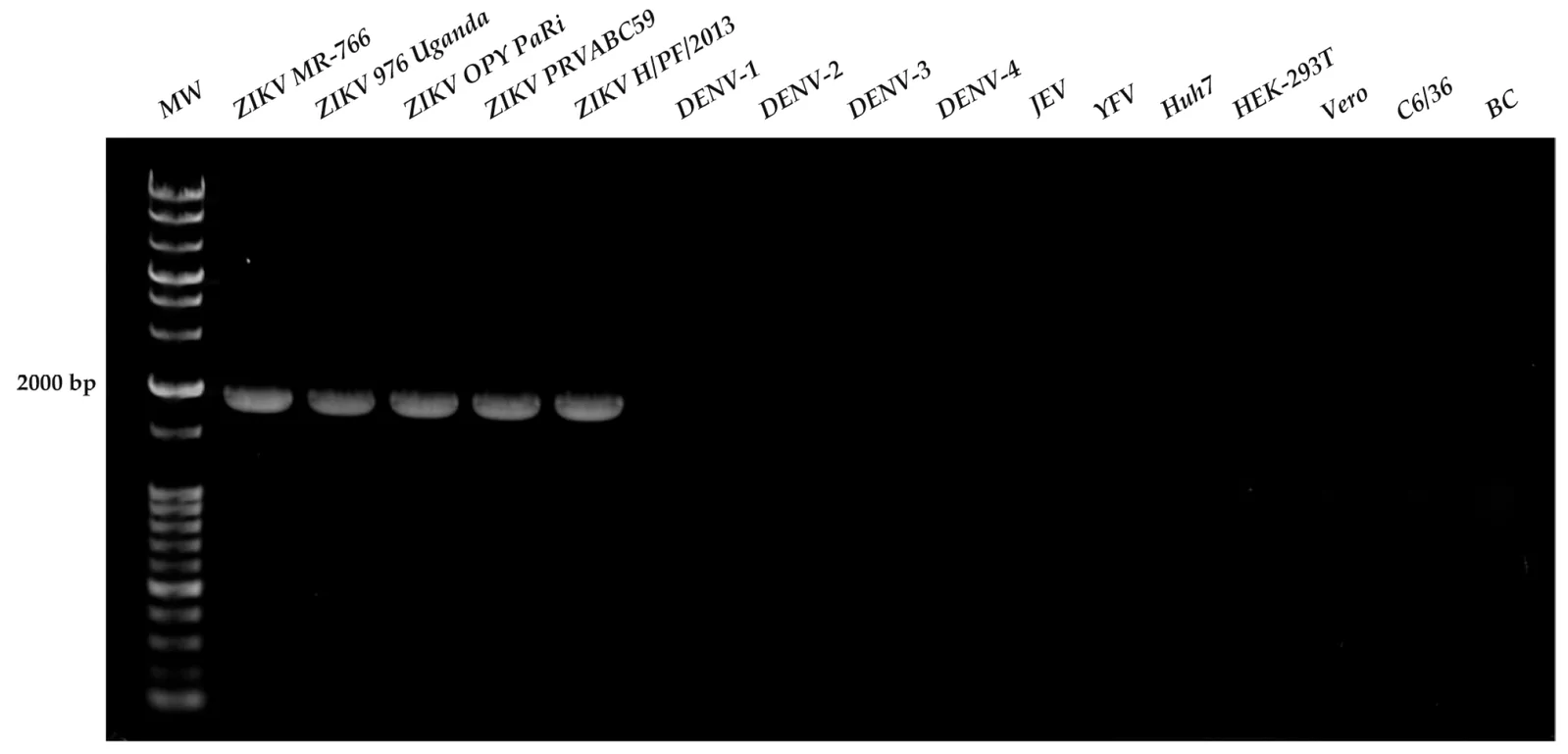
Specificity of the new primer sets for ZIKV detection. MW, molecular weight; ZIKV, Zika virus; DENV, Dengue virus; JEV, Japanese Encephalitis virus; YFV, Yellow Fever virus; Huh7, Huh7 cells; HEK-293T, HEK-293T cells; Vero, Vero cells; C6/36, C6/36 cells; BC, blank control (nuclease-free water); bp, base pair.

Representative ZIKV strains from the African and Asian lineages were used to evaluate primer performance. As shown in Figure 2, the new primers (5F and 1R) successfully detected and amplified the entire E gene in all ZIKV samples tested. Furthermore, no nonspecific amplification was observed with complementary DNA (cDNA) from mammalian cell lines (Huh7, Vero, and HEK-293T), mosquito cells (C6/36), or other flaviviruses [DENV-1–4, Japanese Encephalitis virus (JEV), and Yellow Fever virus (YFV)]. In addition, the primers were independently validated at the Brazil Research Station, Nagasaki University (Pernambuco, Brazil), using the ZIKV PE243 strain isolated during the 2015–2016 ZIKV outbreak in Brazil. The primers successfully amplified the E gene of the Brazilian ZIKV strain (Figure S1).

### 2.3. Sensitivity Analysis

**Figure 3:**
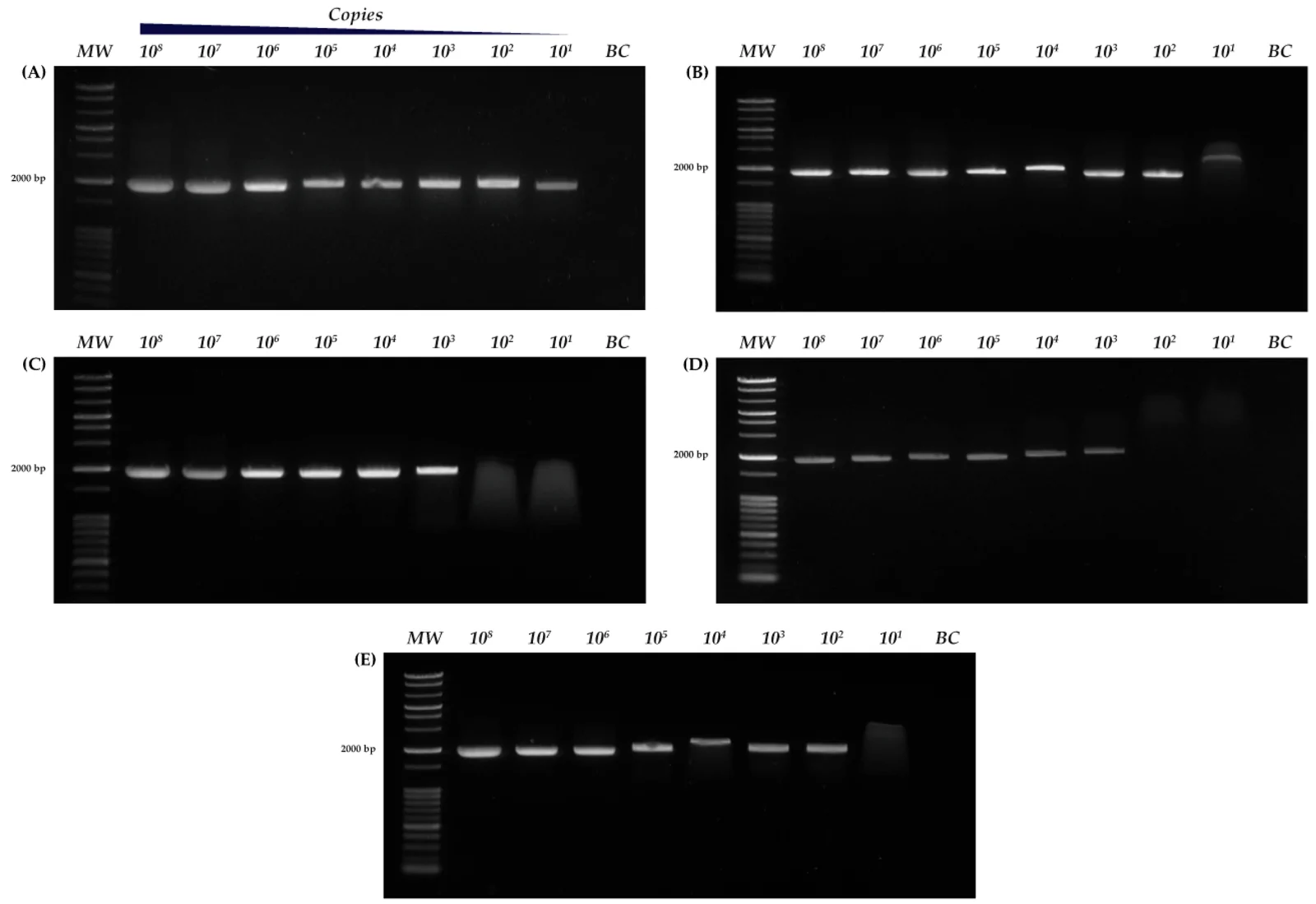
Sensitivity of the newly designed primer sets for ZIKV detection. The detection limits were determined for the five ZIKV strains. Tenfold serial dilutions of ZIKV DNA were prepared and tested by PCR. (**A**) MR-766, (**B**) 976 Uganda, (**C**) MRS OPY PaRi 2015, (**D**) PRVABC59, and (**E**) H/PF/2013. MW, molecular weight; BC, blank control (nuclease-free water); bp, base pair.

The sensitivity of the primers was evaluated using serial ten-fold dilutions of the quantitatively characterized ZIKV E gene DNA amplicon, ranging from 1 × 10^8^ to 1 × 10^0^ copies per reaction. Primers detected the ZIKV genome at low concentrations, with the limit of detection (LOD) ranging from 10^1^ to 10^3^ copies, depending on the viral lineage and strain tested (Figure 3). For African-lineage ZIKV strains (MR-766 and 976 Uganda), the primers detected the viral genome at the lowest concentration tested (10^1^ copies). In contrast, for Asian-lineage strains (MRS OPY PaRi 2015, PRVABC59, and H/PF/2013), the LOD varied among isolates: 10^3^ copies for MRS OPY PaRi 2015 and PRVABC59, and 10^2^ copies for H/PF/2013.

### 2.4. Detection in In Vitro-Spiked Human Samples

**Figure 4:**
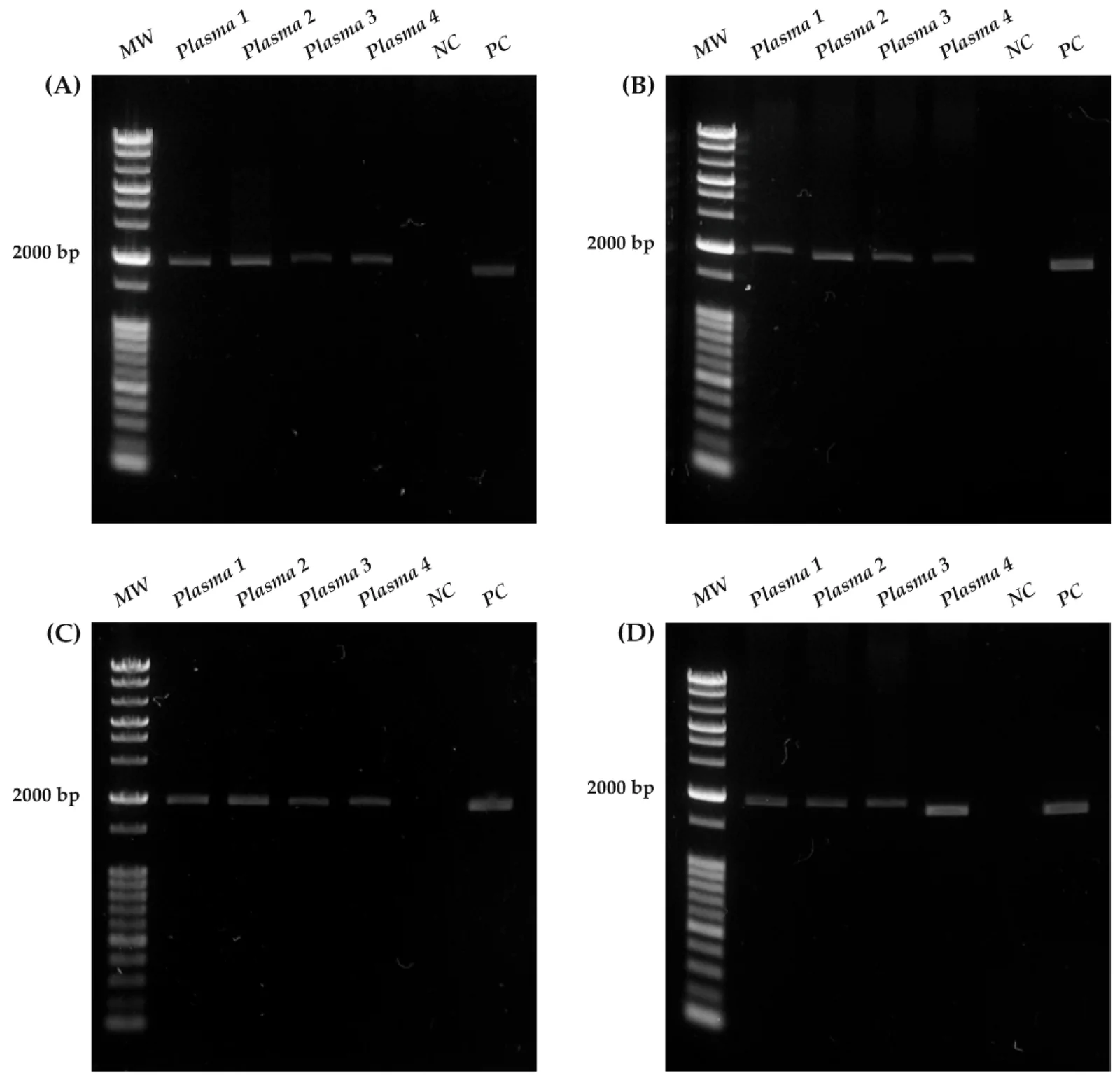
Detection of ZIKV-spiked human plasma samples. (**A**) MR-766, (**B**) MRS OPY PaRi 2015, (**C**) PRVABC59, and (**D**) H/PF/2013. MW, molecular weight; NC, negative control (non-spiked plasma); PC, positive control; bp, base pairs.

The primer sets were evaluated using ZIKV-spiked human plasma samples to assess the potential interference from plasma components. Four plasma samples collected from healthy individuals were used, and ZIKV was spiked at 1 × 10^3^ PFU/mL for each sample (Figure 4). Plasma 1 was collected from an anti-DENV IgG (+) individual, while the other three (plasma 2–4) were collected from DENV-naïve individuals. The primers detected and amplified the ZIKV E gene, even in human plasma. This result suggests that the primers were not negatively affected by plasma components and are suitable for detecting ZIKV in clinical samples.

### 2.5. Sanger Sequencing

All ZIKV samples were sequenced using the six primers designed for this study. Contigs were generated by aligning overlapping sequencing fragments using MAFFT (v7.151) [31], resulting in complete E gene sequences for all ZIKV strain samples, except for the PE243 strain tested in Brazil. The quality of the sequencing data was assessed for each genomic fragment based on raw and trimmed read lengths, mean Phred quality scores, Q20 and Q30 values, and the proportion of ambiguous bases after trimming (Table S2). Overall, the sequencing reads showed good quality after trimming, with only a small proportion of ambiguous base pairs. In addition, the obtained sequences were compared with the corresponding reference genomes to identify nucleotide variations, including single nucleotide polymorphisms and insertions (Table S3). Representative Sanger sequencing chromatograms are shown in Figure S2.

To further assess whether the assay successfully amplified ZIKV isolates representing the relevant genetic lineages, phylogenetic analysis was performed based on the complete E gene sequences generated in this study, together with representative reference sequences from major ZIKV lineages. The resulting phylogenetic tree showed that the sequences generated in this study clustered according to their corresponding genetic lineages, supporting the ability of the primer set to detect and amplify genetically diverse ZIKV strains (Figure S3).

## 3. Discussion

In this study, we developed and validated universal primer sets for amplification and Sanger sequencing of the full-length ZIKV E gene that are applicable to genetically diverse viruses from both the African and Asian lineages. The primer sets consistently amplified all evaluated strains with high analytical specificity and sensitivity, requiring only six primers for complete E gene sequencing. Compared with a previously published protocol [25] (Table S4), our assay was designed using a substantially larger genomic dataset and simplified both amplification and sequencing workflows, providing a practical tool for ZIKV molecular characterization and surveillance.

The broad applicability of the primer sets was demonstrated by their ability to consistently amplify representative viruses from both the African and Asian lineages, despite their substantial genetic diversity. These lineages differ by approximately 12% across the coding region [17,32] and comprise multiple genetically distinct clades circulating worldwide. The successful amplification of representative viruses from the African lineage, as well as the Pacific and American clades of the Asian lineage, indicates that the primer design effectively accommodates this diversity, overcoming an important limitation of assays developed using a limited number of reference genomes.

The absence of non-specific amplification with closely related flaviviruses demonstrated the high specificity of the newly designed primers. This characteristic is particularly important in regions where ZIKV co-circulates with other medically relevant arboviruses that produce similar clinical signs, making laboratory confirmation essential for accurate diagnosis and epidemiological surveillance [33]. Therefore, the high specificity observed in the present study supports the potential utility of these primers for reliable ZIKV detection in arbovirus-endemic areas.

Another strength of this assay is its ability to generate full-length E gene sequences from various ZIKV strains. Because continuous viral evolution may reduce the performance of assays designed using a limited number of reference genomes [34], successful sequencing across distinct lineages indicates that primer design remains robust despite the current ZIKV genetic diversity in Brazil. This capability substantially expands the utility of the assay beyond viral detection, enabling downstream molecular epidemiological studies. To ensure complete coverage of the target region, the outermost primers were positioned 117–282 bp outside the E gene boundaries, and the internal primers generated overlapping sequencing reads. This design minimizes the low-quality regions typically observed at the beginning of Sanger sequencing reads, which are caused by primer-binding artifacts [35]. Importantly, the degenerate positions incorporated into the primers did not result in an apparent loss of sequence quality in the corresponding regions, and overlapping reads provided additional confirmation during contig assembly.

Although WGS provides the highest genomic resolution, its routine implementation is often limited by financial and infrastructure constraints [26]. Under these circumstances, the assay developed here offers a practical alternative by enabling full-length E gene sequencing using the conventional Sanger sequencing methodology. The resulting sequences provide sufficient information for phylogenetic and genomic analyses and are considerably simpler and less expensive than WGS-based approaches [1,24,36]. Moreover, Sanger sequencing continues to provide high accuracy, typically exceeding 99% under optimal conditions [27], making this strategy accessible and reliable for routine surveillance and phylogenetic investigations, particularly in settings where access to next-generation sequencing technologies remains limited.

In addition to its specificity, the assay demonstrated excellent sensitivity for all evaluated strains. Although lower LOD were observed for African-lineage viruses than for Asian-lineage viruses, similar findings have been reported previously [37], suggesting that lineage-specific genetic variation may influence primer annealing efficiency. Nevertheless, all strains were detected within the viral load range commonly observed during acute ZIKV infection (10^3^–10^6^ copies) [38]. Importantly, successful viral detection from ZIKV-spiked human plasma at a concentration of 1 × 10^3^ PFU/mL demonstrated that the assay performance was maintained in a plasma matrix despite the presence of PCR inhibitors commonly found in clinical specimens, including hemoglobin, host nucleic acids, and immunoglobulins [39,40,41]. However, the analytical detection limit was established using serially diluted DNA amplicons and should not be interpreted as the minimum viral concentration detectable in clinical plasma samples. These findings support the potential applicability of the assay to clinical specimens; however, its performance has not yet been evaluated using patient samples. Although direct comparisons are limited by the lack of previous studies evaluating conventional RT-PCR primers targeting the entire ZIKV E gene, the efficiency observed in this study supports the suitability of these primers for the detection and molecular characterization of clinically relevant ZIKV infections.

This study has several limitations. The assay was evaluated using viral isolates and in vitro-spiked human plasma samples, as clinical specimens from naturally infected patients were not available. Although successful amplification was achieved at viral concentrations comparable to those reported during acute ZIKV infection [42,43], the diagnostic performance should be further evaluated using clinical specimens collected from different geographic regions and representing a broader range of viral loads. In addition, although the assay demonstrated high specificity against the flaviviruses evaluated in this study, its specificity should be further assessed using a broader panel of medically relevant flaviviruses, including the West Nile virus (WNV), to further characterize its performance and rule out potential nonspecific amplification.

In conclusion, we developed and validated universal primer sets for complete amplification and Sanger sequencing of the ZIKV E gene, which combine broad lineage coverage with high analytical specificity and sensitivity. By enabling both ZIKV detection and complete E gene sequencing, this assay provides a practical and cost-effective tool for genomic characterization, phylogenetic analysis, molecular epidemiology, and surveillance. Further validation using samples from naturally infected patients and vector-derived samples will strengthen the applicability of the assay for routine molecular epidemiology of genetically diverse ZIKV strains, particularly in resource-limited settings.

## 4. Materials and Methods

### 4.1. Ethics Statement

Because this study involved the use of human plasma samples, this study was submitted and approved by the Research Ethics Committee of the Institute of Tropical Medicine (NEKKEN), Nagasaki University, Japan (230209288, 27 February 2023).

### 4.2. Primers Design

**Figure 5:**
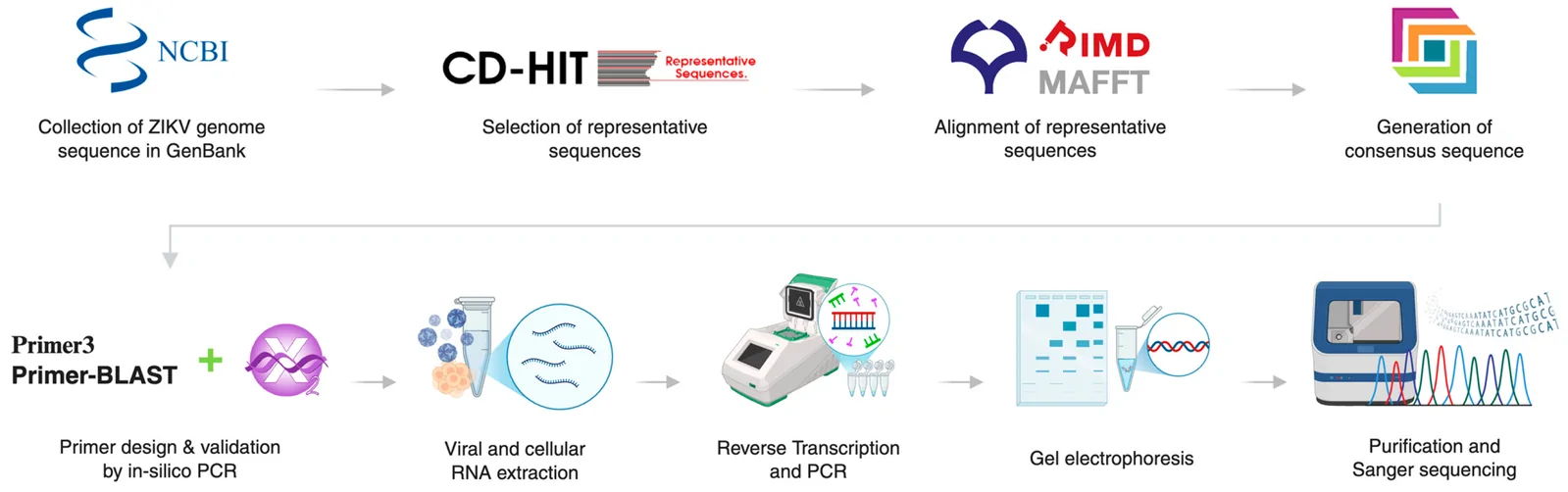
Workflow of primer design and validation, from ZIKV genome sequence collection in public databases to experimental primer evaluation. Created in BioRender. Yuki Takamatsu. (2026) https://BioRender.com/tpawl9k (accessed on 3 July 2026).

All available ZIKV genomic sequences were downloaded from GenBank. Thereafter, the representative sequences were clustered and selected using CD-HIT-EST (v4.8.1) [30] and aligned using MAFFT (v7.151) [31]. The aligned sequences were further analyzed using Jalview (v2.11.4.1) [44] to elaborate the consensus sequence, and degenerate nucleotides were introduced into the primers to cover the variability according to the aligned sequence of each lineage of interest. The primers were designed using Primer3 (v2.6.1) [45] and Primer-BLAST [46] and validated by submitting them for in silico PCR using AmplifX2 (v2.1.1) [47]. A schematic overview of the primer design and validation workflow is shown in Figure 5.

### 4.3. Cells and Viruses

The different ZIKV lineages and isolates of DENV, JEV, and YFV were propagated in either C6/36 (mosquito) or Vero (non-human primate) cells maintained in Gibco^TM^ Minimum Essential Medium (MEM) (Thermo Fisher Scientific, Waltham, MA, USA) supplemented with 2% fetal calf serum (FCS), 50 U/mL penicillin, and 50 µg/mL streptomycin. The cultures were incubated at 28 °C for C6/36 cells and at 37 °C with 5% CO_2_ for Vero cells. ZIKV strains were provided by Prof. Kouichi Morita (Institute of Tropical Medicine, Nagasaki University, Japan) and Dr. Luiz Carlos Alves (Keizo Asami Institute, Federal University of Pernambuco, Brazil). The DENV, JEV, and YFV strain collections were supported by Nagasaki University through the National BioResource Project (NBRP; Human Pathogenic Viruses) of the Ministry of Education, Culture, Sports, Science, and Technology (MEXT), Japan [48]. Huh7 cells were purchased from the Japanese Collection of Research Bioresources (JCRB) Cell Bank (JCRB0403), HEK-293T cells from the American Type Culture Collection (ATCC; CRL-3216), and Vero cells from KAC Co., Ltd. (IU-002), and C6/36 cells were obtained from the European Collection of Authenticated Cell Cultures (ECACC; 89051705). Information on the viruses used in this study is presented in Table 1.

### 4.4. Viral Titration

Viral titers of ZIKV, JEV, and YFV were determined using a plaque assay, whereas DENV infectious titers were determined using a focus-forming assay (FFA), as DENV typically produces diffuse or poorly defined plaques in conventional cell culture systems.

For the plaque assay, Vero cells were seeded in 24-well plates (Thermo Fisher Scientific) at a density of 8 × 10^5^ cells/well and incubated overnight at 37 °C with 5% CO_2_. Virus stocks were serially diluted ten-fold in MEM, and 200 µL of each dilution was inoculated onto the cell monolayers in duplicates. Following 1 h of adsorption at 37 °C, with gentle rocking every 15 min, the cells were overlaid with a semi-solid medium consisting of 1.25% carboxymethylcellulose in MEM supplemented with 2% FCS. The plates were incubated at 37 °C with 5% CO_2_ for 4–7 days, depending on the virus and strain, until visible plaques appeared. The overlay was then removed, and the cells were fixed with 4% paraformaldehyde and stained with 0.25% crystal violet. Plaques were manually counted, and viral titers were expressed as plaque-forming units per milliliter (PFU/mL) and calculated using the following formula: Titer (PFU/mL) = number of plaques ÷ (dilution factor × inoculum volume [mL])

DENV infectious titers were determined using FFA. Vero cells were seeded in 96-well plates (Thermo Fisher Scientific) at a density of 1 × 10^5^ cells/well and incubated overnight at 37 °C with 5% CO_2_. Virus stocks were serially diluted ten-fold in MEM supplemented with 2% FCS, and 100 µL of each dilution was inoculated onto the cell monolayers in duplicate. After 1 h of adsorption at 37 °C, 100 µL of MEM containing 2% FCS and 1.25% methylcellulose was added to each well. The plates were then incubated for 3 days at 37 °C. Cells were subsequently fixed with 10% formalin, permeabilized with 1% NP-40, and blocked with Block-Ace reagent. The monolayers were sequentially incubated with primary and secondary antibodies, each at a 1:1000 dilution, followed by development with DAB substrate. Stained foci were counted microscopically, and the DENV titers were expressed as focus-forming units per milliliter (FFU/mL). Titers were calculated from the mean number of foci in duplicate wells at a countable dilution, adjusted for the dilution factor and 100 µL inoculum volume.

### 4.5. RNA Extraction

Viral RNA extraction was performed using the QIAamp^®^ Viral RNA Kit (QIAGEN, Hilden, Germany) according to the manufacturer’s instructions, and the extracted RNA was stored at −80 °C until further use. Total RNA from mammalian and mosquito cells was extracted using ISOGEN II (Nippon Gene, Toyama, Japan) according to the manufacturer’s instructions.

### 4.6. RT-PCR

The extracted viral and cellular RNAs were subjected to reverse transcription (RT) using the ReverTra Ace^®^ kit (Toyobo, Osaka, Japan) for complementary DNA (cDNA) synthesis. The reaction was carried out in a total volume of 20 µL, containing 4 µL of 5× RT Buffer, 2 µL of dNTP mixture (10 mM), 0.5 µL of RNase inhibitor (10 U/µL), 1 µL of random primers (25 pmol/µL), 1 µL of ReverTraAce (100 U/µL), and approximately 500 ng of extracted RNA. RNA samples were denatured at 65 °C for 5 min, followed by immediate incubation on ice for 1 min before adding to the reaction mixture. The reaction was performed using an Applied Biosystems^TM^ SimpliAmp^TM^ Thermal Cycler (Thermo Fisher Scientific) under the following conditions: (1) annealing at 30 °C for 10 min; (2) enzymatic reaction at 42 °C for 30 min; and (3) denaturation at 99 °C for 5 min. Following the reaction, cDNA concentration and quality were analyzed using a NanoDrop^TM^ ND-1000 spectrophotometer (Thermo Fisher Scientific).

PCR amplification was performed using KOD One^®^ PCR Master Mix (Toyobo) in a total volume of 50 µL. The reaction mixture consisted of 25 µL of 2× KOD One^®^ PCR Master Mix, 1.5 µL of each newly designed amplification primer (10 µM), and approximately 50 ng of cDNA. Amplification consisted of 30 cycles of denaturation at 98 °C, annealing at 60 °C, and elongation at 68 °C, each for 10 s. The PCR products were loaded onto a 1% agarose gel, subjected to electrophoresis at 100 V for 30 min, and the results were analyzed under light-emitting diode (LED) light.

The reaction also included DNA samples from mammalian and mosquito cells and other flaviviruses (DENV, JEV, and YFV) to assess primer specificity.

For the analytical sensitivity evaluation, a DNA standard containing a defined ZIKV E gene sequence was used. DNA concentration was determined using a Nanodrop^TM^ ND-1000 spectrophotometer (Thermo Fisher Scientific), and the copy number was calculated based on the molecular weight and length of the DNA fragment. The DNA standard was then used to generate a quantitative standard curve by qPCR, with each standard concentration being analyzed in duplicate. ZIKV E gene amplicons generated from the evaluated strains were quantified by qPCR using a standard curve to determine their copy numbers. The quantified amplicons were adjusted to a starting concentration of 1 × 10^8^ DNA copies and serially diluted ten-fold. Each dilution was subsequently tested in duplicate by endpoint PCR to determine the analytical detection limit of the primer set.

Bands from positive samples were excised and purified from the gel using the Wizard^®^ SV Gel and PCR Clean-Up System kit (Promega, WI, USA) according to the manufacturer’s instructions. The recovered DNA was used as a template for sequencing to evaluate the newly designed sequencing primers.

### 4.7. Sequencing

Sequence PCR was performed using the QuantumSeq^TM^ QuantumDye^TM^ Terminator v3.1 Cycle Sequencing Kit (TOMY Digital Biology, Tokyo, Japan) in a total volume of 10 µL consisting of 1 µL of 2.5× QuantumDye^TM^ Cycle Sequencing Mix, 2 µL of 5× QuantumDye^TM^ Sequencing Buffer, 1 µL of primer (3.2 µM), and approximately 100 ng of DNA template. The reaction consisted of denaturation at 96 °C for 1 min, followed by 25 cycles at 96 °C for 10 s, 60 °C for 5 s, and 60 °C for 1 min.

The sequence PCR product was purified using the Agencourt^®^ CleanSEQ^®^ Dye-Terminator Removal kit (Beckman Coulter, CA, USA) according to the manufacturer’s instructions to remove unincorporated dyes, nucleotides, salts, and contaminants from the PCR products. After purification, the samples were sequenced using an Applied Biosystems^TM^ 3500 Genetic Analyzer (Thermo Fisher Scientific), and the results were analyzed using Phred (v0.020425) [50] and Phrap (v0.990319) [51] software to generate contigs for each ZIKV isolate. All primers used in this study are listed in Table 2.

### 4.8. Plasma Samples

To evaluate the performance of the primers for ZIKV detection in human biological samples, plasma from four healthy individuals was spiked with ZIKV supernatant and subjected to RNA extraction, followed by RT-PCR. Plasma samples were obtained from healthy adult volunteers.

## Figures and Tables

**Table 1:** Viruses used to determine the specificity and sensitivity of RT-PCR.

Virus	Lineage (Clade)/Genotype	Strain	Location	Accession Number
ZIKV	African	MR-766	Uganda	LC002520
	African	976 Uganda	Uganda	Available only in EVA [49]
	Asian (Pacific)	MRS OPY PaRi 2015	Martinique	KU647676
	Asian (Pacific)	H/PF/2013	French Polynesia	KJ776791
	Asian (American)	PRVABC59	Puerto Rico	KU501215
	Asian (American)	PE243	Brazil	KX197192
DENV-1	IV	99st12A	Philippines	MW793679
DENV-2	Cosmopolitan	Sr-100/17	Sri Lanka	MN083210
DENV-3	I	YCH228	Myanmar	MW788885
DENV-4	I	SLMC318	Philippines	PP422236
JEV	III	JaOArS982	Japan	M18370
YFV	17D Vaccine strain		Ghana	MT107250

ZIKV, Zika virus; DENV, Dengue virus; JEV, Japanese Encephalitis virus; YFV, Yellow Fever virus.

**Table 2:** Sequences of the newly designed primers used in this study.

Primer ID	Sequence (5′–3′)	Position ^1^
ZIKV.E_5F *	TGGGTYGTGTACGKAACCTG	695–714
ZIKV.Eseq_1a	ATGTCAGGYGGGACYTGGGT	1019–1038
ZIKV.Eseq_2b	GGAGTAYCGGATAATGYTRTCAG	1381–1403
ZIKV.Eseq_3a	GGCYGAGATGGATGGWGCAA	1798–1817
ZIKV.Eseq_4a	TTYGARGCCACTGTGAGAGGYG	2206–2227
ZIKV.E_1R *	RGGRGAGTCRGGATGGTACT	2584–2603

^1^ Position based on the consensus sequence; * Primers used for full-length E gene amplification.

## Data Availability

The original contributions presented in this study are included in this article. The consensus sequences of all ZIKV strains generated using the newly designed primers are available as FASTA files in the Supplementary Materials. Further inquiries should be directed to the corresponding author.

## Supplementary Materials

The following supporting information can be downloaded at: https://www.mdpi.com/article/10.3390/ijms27188017/s1.
