# Supplementary material for: Development of Universal Primer Sets for Zika Virus Envelope Gene Amplification and Sequencing
Source: Int J Mol Sci. 2026 Sep 9;27(18):8017. doi: 10.3390/ijms27188017 (PMC13607635; doi:10.3390/ijms27188017)
Supplement: Supplementary file 1 [file ijms-27-08017-s001.zip › Figure S1.pdf]

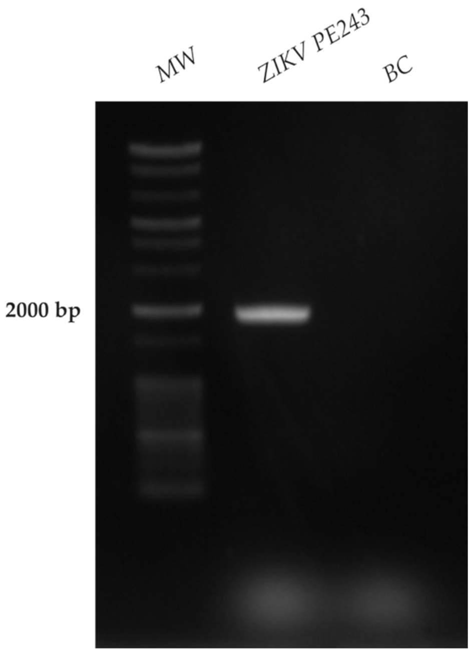

Figure S1. Detection of the ZIKV PE243 strain using new primer sets. MW, molecular weight; ZIKV, Zika virus; BC, blank control (nuclease-free water); bp, base pair.
