# Supplementary material for: Development of Universal Primer Sets for Zika Virus Envelope Gene Amplification and Sequencing
Source: Int J Mol Sci. 2026 Sep 9;27(18):8017. doi: 10.3390/ijms27188017 (PMC13607635; doi:10.3390/ijms27188017)
Supplement: Supplementary file 1 [file ijms-27-08017-s001.zip › Figure S2.pdf]

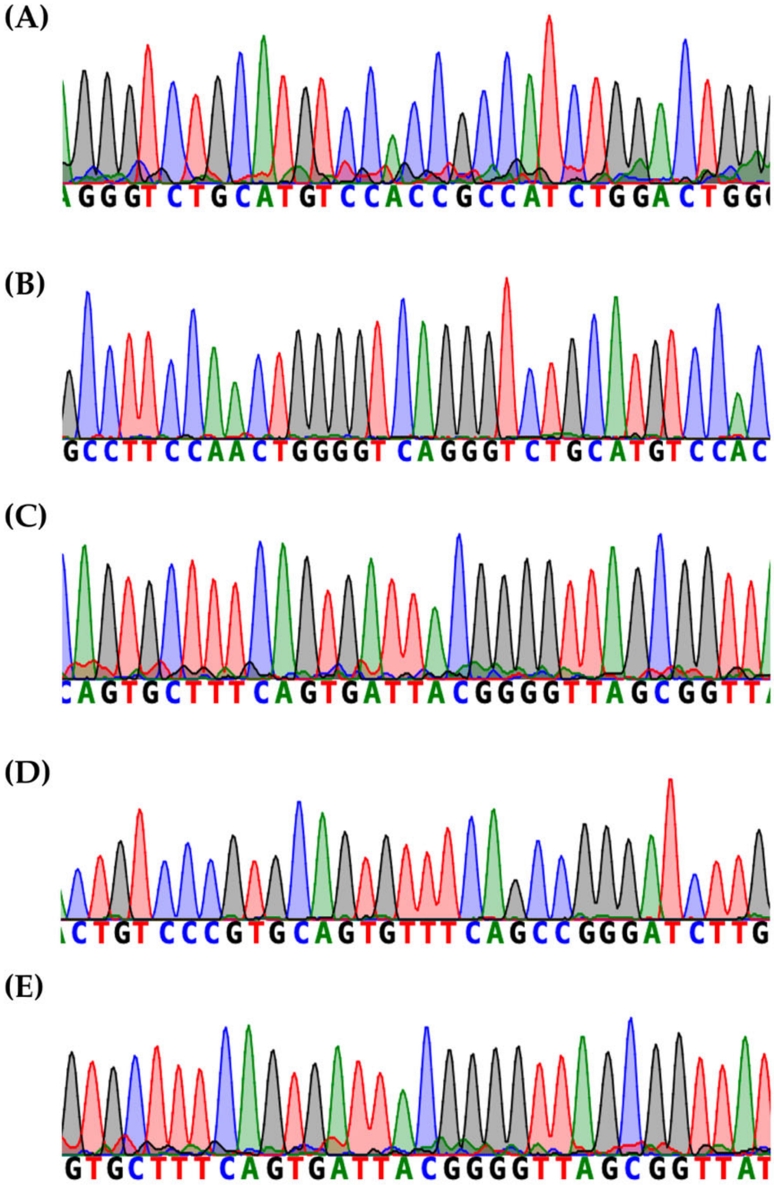

Figure S2. Representative Sanger sequencing chromatograms obtained using the newly designed ZIKV sequencing primers. (A) MR-766; (B) 976 Uganda; (C) MRS OPY PaRi 2015; (D) PRVABC59; (E) H/PF/2013.
