# Supplementary material for: Development of Universal Primer Sets for Zika Virus Envelope Gene Amplification and Sequencing
Source: Int J Mol Sci. 2026 Sep 9;27(18):8017. doi: 10.3390/ijms27188017 (PMC13607635; doi:10.3390/ijms27188017)
Supplement: Supplementary file 1 [file ijms-27-08017-s001.zip › Figure S3.pdf]

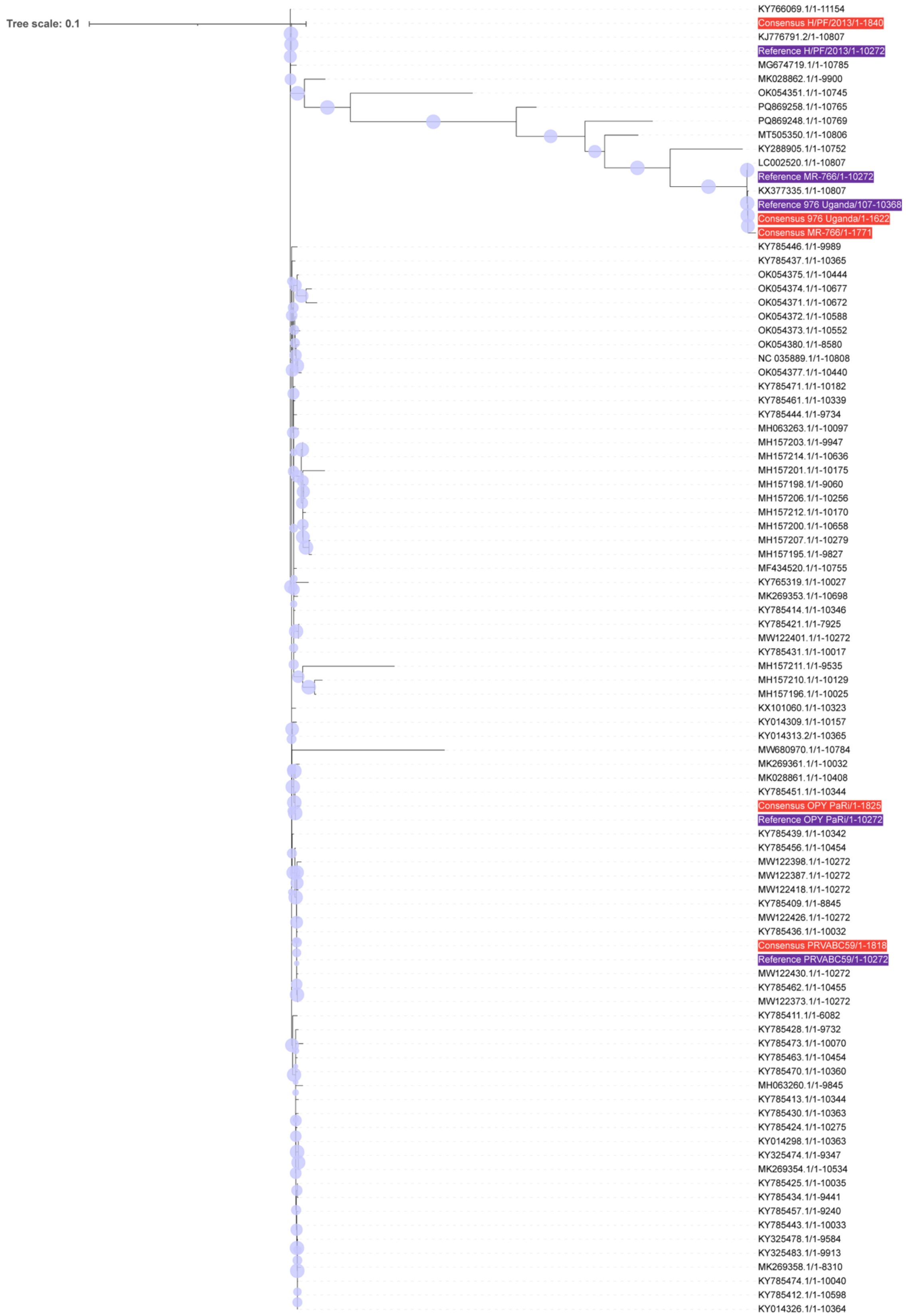

Figure S3. Comparison of the reference ZIKV sequence with the consensus sequence generated using the newly developed primers. The reference sequence is shown in purple, whereas the consensus sequence generated in this study using the newly developed primers is shown in red.
