# Supplementary material for: Development of Universal Primer Sets for Zika Virus Envelope Gene Amplification and Sequencing
Source: Int J Mol Sci. 2026 Sep 9;27(18):8017. doi: 10.3390/ijms27188017 (PMC13607635; doi:10.3390/ijms27188017)
Supplement: Supplementary file 1 [file ijms-27-08017-s001.zip › Table S3.pdf]

Table S3. Sequence variations identified in ZIKV isolates compared with the reference genome.

Isolate	Position	Alteration Type	Reference	Alteration
MR-766	1100	SNP	T	C
	1432	SNP	C	A
	1444	SNP	A	T
	1445	SNP	T	G
	1446	SNP	A	G
	1671	SNP	T	C
	1772	SNP	T	G
	1894	INS	-	C
	2077	INS	-	A
	2103	INS	-	T
	2137	INS	-	T
	2176	INS	-	G
	2401	SNP	T	A
	2503	SNP	T	C
	2540	INS	-	G
	2558	INS	-	T
976 Uganda	-	-	-	-
H/PF/2013	1752	INS	-	G
PRVABC59	-	-	-	-
OPY PaRi	1219	INS	-	G
	1252	SNP	A	G
	1253	SNP	G	A
	1253	INS	-	GC
	1255	SNP	C	T
	1256	SNP	T	G
	1287	INS	-	A
	1296	INS	-	G
	2390	INS	-	A

The table lists the genomic position, type of nucleotide alteration, and the corresponding reference and altered nucleotides for each ZIKV isolate. SNP, single-nucleotide polymorphism; INS, insertion. A dash (–) indicates that no nucleotide variation was identified at the corresponding position.
