# Supplementary material for: Development of Universal Primer Sets for Zika Virus Envelope Gene Amplification and Sequencing
Source: Int J Mol Sci. 2026 Sep 9;27(18):8017. doi: 10.3390/ijms27188017 (PMC13607635; doi:10.3390/ijms27188017)
Supplement: Supplementary file 1 [file ijms-27-08017-s001.zip › Table S4.pdf]

Table S4. Comparison of published protocols for complete ZIKV E gene amplification and sequencing.

Study	Representative sequences used for primer design (n)	Samples used for experimental validation		Primers required for complete E gene sequencing (n)	Analytical sensitivity (LOD)	Primer validation
		Geographic representation	ZIKV lineages (clade) represented			
[25]	7	Brazil	Asian (American)	8	Not reported	1) Isolated virus detection 2) Sequencing
Present study	135	Uganda/Martinique/French Polynesia/Puerto Rico/Brazil	African and Asian (Pacific and American)	6	10^1-10^3 copies	1) *In silico* PCR 2) Isolated virus detection 3) Serial dilution 4) ZIKV-spiked human plasma 5) Sequencing

ZIKV, Zika virus; E, Envelope gene; LOD, Limit of detection.
